# Sox2 Uses Multiple Domains to Associate with Proteins Present in Sox2-Protein Complexes

**DOI:** 10.1371/journal.pone.0015486

**Published:** 2010-11-12

**Authors:** Jesse L. Cox, Sunil K. Mallanna, Xu Luo, Angie Rizzino

**Affiliations:** 1 Eppley Institute for Research in Cancer and Allied Diseases, University of Nebraska Medical Center, Omaha, Nebraska, United States of America; 2 Department of Biochemistry and Molecular Biology, University of Nebraska Medical Center, Omaha, Nebraska, United States of America; The Hong Kong University of Science and Technology, People's Republic of China

## Abstract

Master regulators, such as Sox2, Oct4 and Nanog, control complex gene networks necessary for the self-renewal and pluripotency of embryonic stem cells (ESC). These master regulators associate with co-activators and co-repressors to precisely control their gene targets. Recent studies using proteomic analysis have identified a large, diverse group of co-activators and co-repressors that associate with master regulators, including Sox2. In this report, we examined the size distribution of nuclear protein complexes containing Sox2 and its associated proteins HDAC1, Sall4 and Lin28. Interestingly, we determined that Sox2 and HDAC1 associate with protein complexes that vary greatly in size; whereas, Lin28 primarily associates with smaller complexes, and Sall4 primarily associates with larger complexes. Additionally, we examined the domains of Sox2 necessary to mediate its association with its partner proteins Sall4, HDAC1 and HDAC2. We determined that Sox2 uses multiple and distinct domains to associate with its partner proteins. We also examined the domains of Sox2 necessary to mediate its self-association, and we determined that Sox2 self-association is mediated through multiple domains. Collectively, these studies provide novel insights into how Sox2 is able to associate with a wide array of nuclear proteins that control gene transcription.

## Introduction

Embryonic stem cells (ESC) are able to self-renew or differentiate into cells from each of the three embryonic germ layers. The growth and differentiation of ESC is regulated by complex gene regulatory networks under the control of a growing list of transcription factors that behave as master regulators. Three transcription factors in particular, Sox2, Oct4 (also known as Oct3, Oct3/4 and Pou5f1) and Nanog, have been shown to form an essential core of the transcriptional machinery required for the self-renewal and pluripotency of ESC [1]. Moreover, each of these transcription factors has been shown to be essential for normal embryonic development [2]–[4]. As expected for master regulators, Sox2, Oct4 and Nanog have been shown to regulate the expression of other essential genes, as well as their own transcription by both positive and negative feedback loops [5]–[9].

Efforts to understand how Sox2, Oct4 and Nanog mediate their effects in ESC have included genome-wide DNA binding studies [6], [10] and, more recently, proteomic screens to identify nuclear proteins that associate with these master regulators [11]–[15]. Interestingly, the latter studies indicate that Sox2, Oct4 and Nanog associate with a wide array of co-activators and co-repressors. Using an unbiased proteomic screen, our laboratory recently identified a wide array of Sox2-associated proteins, including members of the transcriptionally repressive NuRD complex, such as histone deacetylase (HDAC) 1, as well as transcription factors, such as Sall4, and RNA binding proteins, such as Lin28 [15]. Proteomic analyses conducted by others have provided details regarding the Nanog- and Oct4-interactomes. Remarkably, these reports indicate that Sox2, Oct4 and Nanog associate with many of the same proteins. For example, Oct4, Nanog and Sox2 have each been shown to associate with Brg1, HDAC1 and Sall4, which are required for the self-renewal and pluripotency of ESC [11]–[15].

The identification of proteins that associate with master regulators raised a number of important questions, including: are master regulators present in multiple, diverse protein complexes; how do these protein associations affect the function of master regulators; and, which domains of master regulators are required for protein-protein association? Efforts to address these questions have begun, and some progress has been made. Fractionation of nuclear extracts prepared from ESC has shown that Nanog is present in complexes that vary in size from 160 kDa to 1 MDa [11], [12]. Although further study is needed, it is highly likely that different Nanog-protein complexes contribute to the wide range of different cellular processes required for ESC self-renewal and pluripotency. Thus far, the size distribution of Oct4- and Sox2-protein complexes has not been reported.

In this study, we have begun to address some of the questions discussed above with regard to Sox2-protein complexes. For this purpose, we initially examined the size distribution of Sox2-protein complexes using ESC. These studies indicated that Sox2 is present in distinct protein complexes that vary considerably in size. Given our recent finding that Sox2 associates with >60 nuclear proteins in ESC undergoing differentiation [15], we tested the hypothesis that Sox2 uses different domains to associate with its different protein partners. For this purpose, we mapped the domains of Sox2 required for its association with several proteins, Sall4, HDAC1 and HDAC2, found in Sox2-protein complexes. Moreover, we mapped the domains used by Sox2 that enable it to associate with itself.

## Results

### Sox2 is present in multiple protein complexes

**Figure 1:**
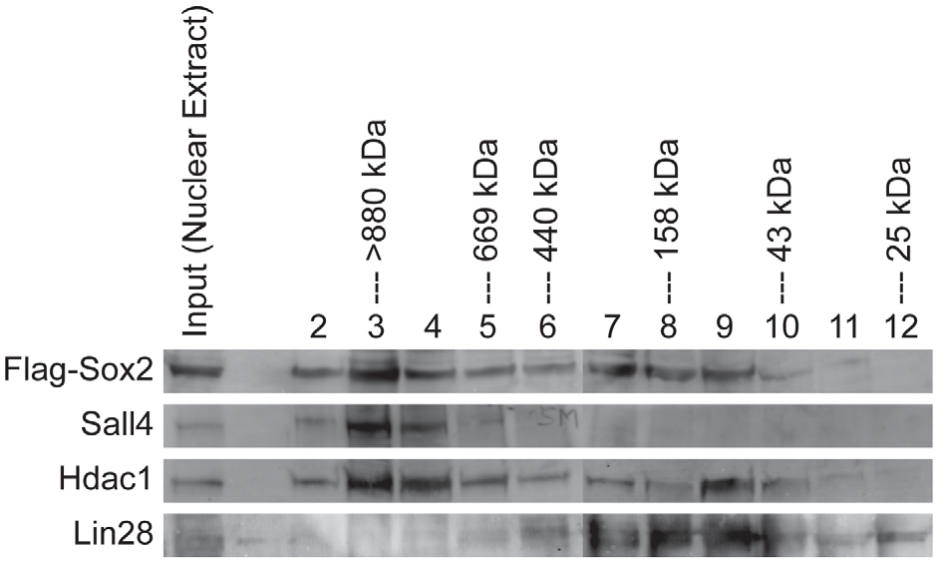
Fractionation of nuclear protein complexes from ESC. Nuclear proteins were isolated from dox-induced mouse ESC engineered to express Flag-Sox2 from a dox-inducible transgene. Nuclear proteins were then size fractionated using a Superdex™-200 column under non-denaturing conditions. Fractions were concentrated and western blot analyses were conducted using the indicated antibodies: α-Sox2 (top), α-Sall4 (upper-middle), α-HDAC1 (lower-middle), or α-Lin28 (bottom), as described in the Materials and Methods.

Our unbiased proteomic screen of Sox2-associated protein revealed that Sox2 associates with >60 nuclear proteins [15]. We initiated the studies in this report by examining the size distribution of Sox2-protein complexes. For this purpose, we used mouse ESC that were previously engineered to express an epitope-tagged form of Sox2 (Flag-Sox2) from a dox-inducible transgene [16], which are referred to as inducible-Sox2-ESC (i-Sox2-ESC). One day after treatment with doxycycline (dox), which elevated the levels of Sox2 approximately 2-fold above that in untreated ESC, nuclear extracts were prepared from the cells. Nuclear proteins were size fractionated using a SuperdexTM-200 column under non-denaturing conditions. Proteins in the different chromatographic fractions were concentrated and probed initially by western blot analysis for Sox2. Sox2 protein was detected over a size distribution ranging from ∼40 kDa to >800 kDa (Figure 1), arguing that Sox2 is present in multiple protein complexes.

We extended this analysis by examining the distribution of proteins known to both associate with Sox2 and influence the fate of mouse ESC. HDAC1 was found in protein fractions throughout the sizes examined, although HDAC1 was enriched as free form (∼65 kDa) and in large protein complexes (>670 kDa) (Figure 1). In contrast, Sall4 was primarily detected in protein complexes >670 kDa; whereas, Lin28 was found primarily in smaller protein complexes <440 kDa in size. Thus, although Sall4, HDAC1 and Lin28 have been shown to associate with Sox2, the overall size distributions of Sall4 and Lin28 differ from that of Sox2.

### Sox2 uses at least two domains to associate with Sall4

**Figure 2:**
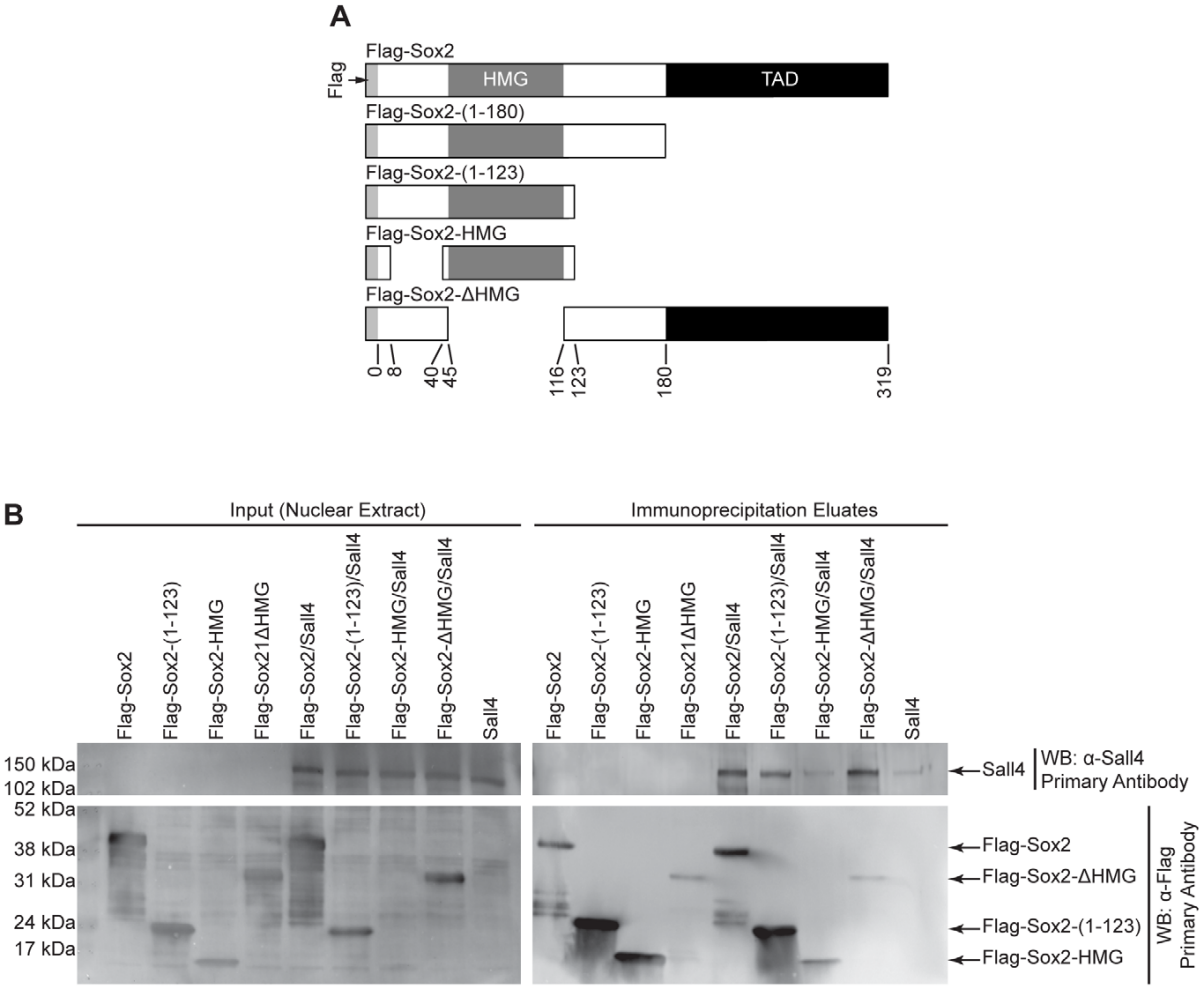
Domains of Sox2 used for association with Sall4. (A) Schematic diagrams of the Flag-Sox2 expression constructs used for domain mapping studies. (B) Mapping domains of Sox2 that mediate its association with Sall4. 293T cells were transiently transfected with an expression construct for Sall4 and the Sox2 constructs shown, and nuclear extracts were prepared 1 day later. Nuclear extracts (input lanes) used for co-immunoprecipitation of Sall4 are presented as western blot analyses, probing for Sall4 (top-left) or the Flag-Sox2 constructs indicated (bottom-left). M2-beads were used to co-immunoprecipitate Flag-Sox2 constructs and their associated proteins. Immunoprecipitate eluates were used in western blot analysis (right panels), and were probed for either Sall4 (top-right) or the Flag-Sox2 constructs (bottom-right). These experiments were repeated, and similar results were observed.

Because Sox2 associates with numerous proteins, it is highly likely that Sox2 uses separate domains to associate with different proteins. We initially tested this possibility by mapping the domains of Sox2 necessary to co-immunoprecipitate the Sox2-associated protein, Sall4. For this purpose, we generated expression plasmids for different Flag-Sox2 deletion constructs. These constructs express modified Flag-Sox2 proteins: Flag-Sox2-HMG that contains only the DNA binding domain, Flag-Sox2-(1-123) that contains the N-terminal and the DNA-binding domains, or Flag-Sox2-ΔHMG that lacks the DNA binding domain (Figure 2A).

For our domain mapping studies, 293T cells were transiently transfected with expression plasmids for Flag-Sox2 or one of the Flag-Sox2 deletion mutants, together with an expression plasmid for Sall4. Although a small amount of Sall4 was non-specifically pulled down by α-Flag M2 affinity beads (M2-beads), there was substantially more Sall4 co-immunoprecipitated when the cells were transfected with both the full-length Sox2 and the Sall4 expression constructs. These findings argue that Sox2 associates with Sall4, not only in ESC [15], but also in a heterologous system (Figure 2B). We determined that Sall4 is also co-immunoprecipitated by Flag-Sox2-(1-123). However, the amount of Sall4 co-immunoprecipitated by Flag-Sox2-(1-123) was less than that co-immunoprecipitated by full-length Flag-Sox2, even though the amount of Flag-Sox2-(1-123) was substantially greater than full-length Flag-Sox2 in the co-immunoprecipitation eluate. This argues that removal of the region C-terminal to the HMG domain of Sox2 decreases its association with Sall4. Consistent with this result, Flag-Sox2-ΔHMG, which contains the C-terminal as well as the N-terminal domains of Sox2, successfully co-immunoprecipitated Sall4 even though its amount in the immunoprecipitation eluate was the lowest among all the different Flag-Sox2 proteins used in this study. We also determined that Flag-Sox2-HMG co-immunoprecipitated little if any Sall4. We do not believe that this is simply due to the low amount of Flag-Sox2-HMG present in the co-immunoprecipitation eluate, given the large amount of Sall4 co-immunoprecipitated by Flag-Sox2-ΔHMG, which had the lowest expression of the Sox2 constructs in this experiment. Although equal amounts of proteins were loaded for western blot analysis, as evidenced by uniform levels of Sall4, the levels of Flag-Sox2 constructs in the input likely differ because of inherent differences in the stability of Flag-Sox2 protein constructs.

Together, these findings lead to the conclusion that Sox2 and Sall4 associate with one another primarily through a Sox2 domain that is C-terminal to its HMG domain, and to a lesser extent, a domain N-terminal to the Sox2-HMG domain. Nonetheless, we cannot completely rule out the possibility that the HMG domain of Sox2 also promotes the association with Sall4. The diminished ability of Sall4 to be co-immunoprecipitated by Flag-Sox2-HMG and the Flag-Sox2-(1-123) may be due to slight misfolding of the protein. However, this seems unlikely. As discussed below, Flag-Sox2-HMG and Flag-Sox2-(1-123) promote the association with other Sox2-associated proteins. Equally important, the N-terminal domain of Sox2 (amino acids 1 to 123), previously shown to associate with Oct4, was used to determine the crystal structure of the HMG domain of Sox2 [17].

### HDAC1 and HDAC2 are co-immunoprecipitated by different Flag-Sox2 constructs

**Figure 3:**
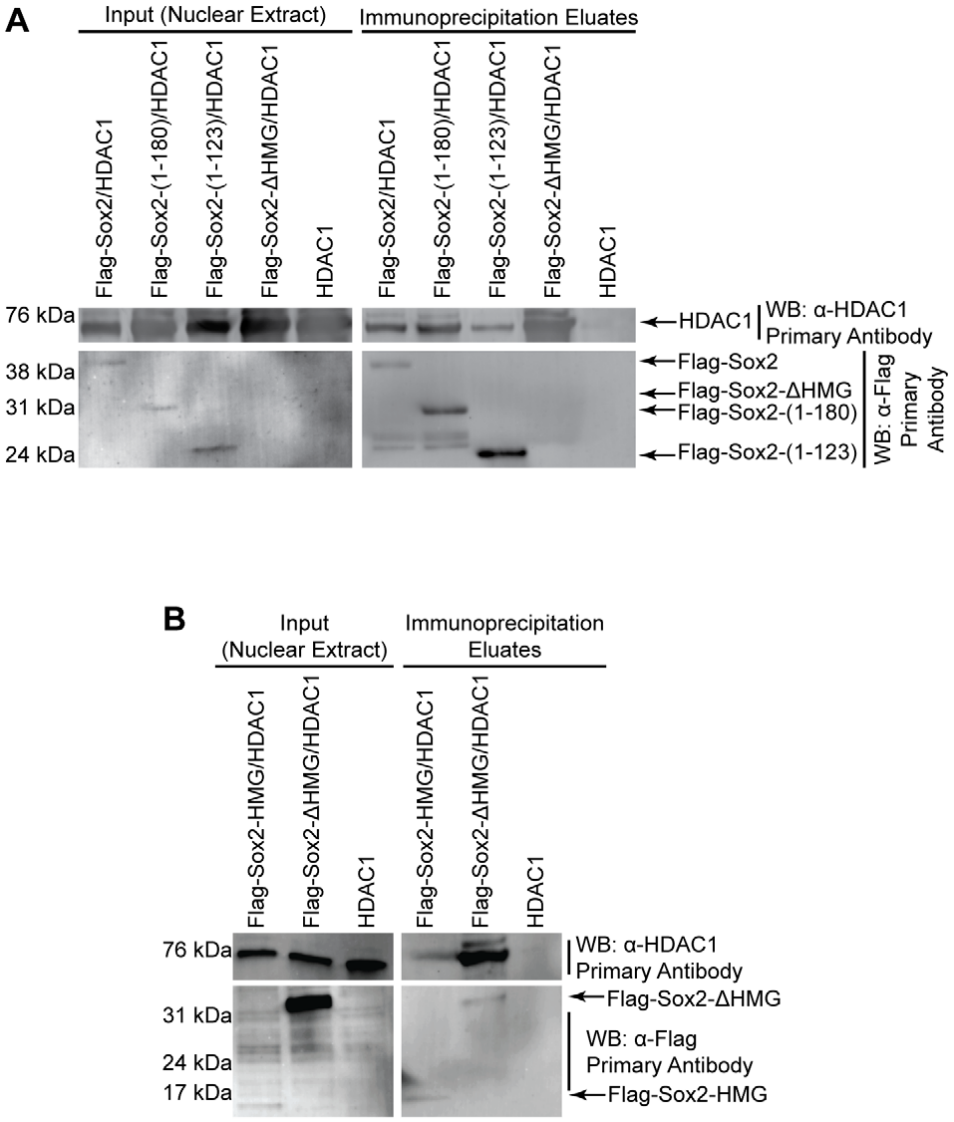
Domains of Sox2 used for association with HDAC1. (A) Mapping domains of Sox2 that mediate its association with HDAC1. 293T cells were transiently transfected with an expression construct for HDAC1 and the Sox2 constructs shown, and nuclear extracts were prepared 1 day later. Nuclear extracts (input lanes) used for co-immunoprecipitation of HDAC1 are presented as western blot analyses, probing for HDAC1 (top-left) or the Flag-Sox2 constructs indicated (bottom-left). M2-beads were used to co-immunoprecipitate the Flag-Sox2 constructs and their associated proteins. Immunoprecipitate eluates were used for western blots (right panels), and were probed for either HDAC1 (top-right) or the Flag-Sox2 constructs (bottom-right). (B) Co-immunoprecipitation of HDAC1 by Flag-Sox2-HMG or Flag-Sox2-ΔHMG. Experimental design was the same as described for Figure 3A. The experiments described were repeated, and similar results were observed.

Next, we mapped the domains of Sox2 required for its association with HDAC1. Initially, we determined that full-length Flag-Sox2 co-immunoprecipitated HDAC1 in 293T cells (Figure 3A). As a control, we determined that HDAC1 was not detected in the co-immunoprecipitation eluate from the cells transfected with the expression plasmid for HDAC1 alone. Interestingly, a different Flag-Sox2 construct that omits the transactivation domain of Sox2 [Flag-Sox2-(1-180)] (Figure 2A), was able to associate with HDAC1 as robustly as full-length Flag-Sox2 (Figure 3A). We further characterized the co-immunoprecipitation of HDAC1 with our Flag-Sox2-(1-123) construct. As noted above for Sall4, the amount of HDAC1 co-immunoprecipitated by Flag-Sox2-(1-123) was significantly less than the amount co-immunoprecipitated by full-length Flag-Sox2, even though Flag-Sox2-(1-123) was present in the co-immunoprecipitation eluate at amounts greater than the full-length Flag-Sox2. Therefore, the region of Sox2 between amino acids 123 to 180 appears to mediate the association between Sox2 and HDAC1. Interestingly, deletion of the HMG domain in the context of full-length Sox2 (Flag-Sox2-ΔHMG) dramatically increased the amount of HDAC1 able to associate with Sox2, even though Flag-Sox2-ΔHMG was below the limit of detection. Additionally, Flag-Sox2-HMG co-immunoprecipitated less HDAC1 than did Flag-Sox2-ΔHMG (Figure 3B). Thus, it appears that multiple domains of Sox2 contribute to its association with HDAC1, and the HMG domain, in the context of full-length Sox2, interferes with this association.

**Figure 4:**
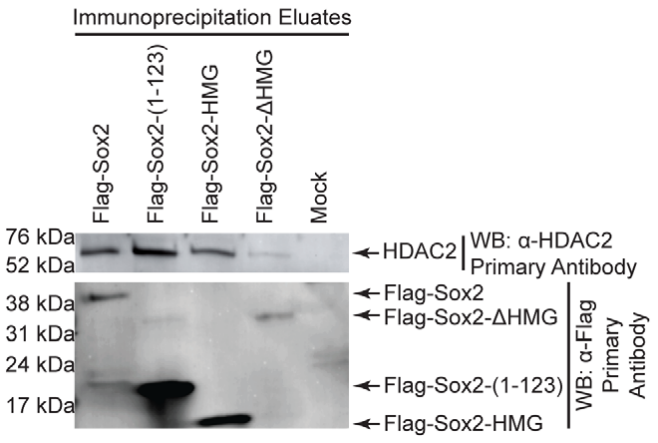
Co-immunoprecipitation of endogenous 293T cell HDAC2 by exogenous Flag-Sox2 constructs. The indicated constructs were transiently transfected into 293T cells, and nuclear proteins were prepared 1 day later. Flag-Sox2 proteins and associated proteins were co-immunoprecipitated from nuclear extracts using M2-beads. Immunoprecipitate eluates were used in western blot analyses, and were probed for either α-HDAC2 (top) or α-Flag (bottom). Protein for the control (mock) lane was from un-transfected 293T cells. This experiment was repeated, and similar results were observed.

Our laboratory and others have demonstrated that Sox2 also associates with HDAC2 [15], [18]. Therefore, we examined the domains of Sox2 required to associate with HDAC2. For this purpose, we transfected 293T cells with the expression plasmid for Flag-Sox2 and determined that Flag-Sox2 is able to co-immunoprecipitate endogenous HDAC2 (Figure 4). HDAC2 was not detected in the co-immunoprecipitation eluate from cells containing only endogenous HDAC2, which served as a negative control. This enabled us to map the domains of Sox2 required for its association with HDAC2 endogenously expressed by 293T cells (Figure 4). We determined that full-length Flag-Sox2, Flag-Sox2-(1-123), and Flag-Sox2-HMG each successfully co-immunoprecipitated endogenous HDAC2. Interestingly, the amount of HDAC2 co-immunoprecipitated by Flag-Sox2-(1-123) was greater than the amount co-immunoprecipitated by either full-length Flag-Sox2 or Flag-Sox2-HMG. This was most likely due to high amounts of Flag-Sox2-(1-123) in the co-immunoprecipitation eluate compared to amounts of Flag-Sox2 and Flag-Sox2-HMG. Additionally, we determined that Flag-Sox2-ΔHMG failed to pull-down endogenous HDAC2. Together, these results argue that Sox2 primarily uses a single domain, the HMG domain, for its association with HDAC2.

### Sox2 self-association is mediated by multiple domains

**Figure 5:**
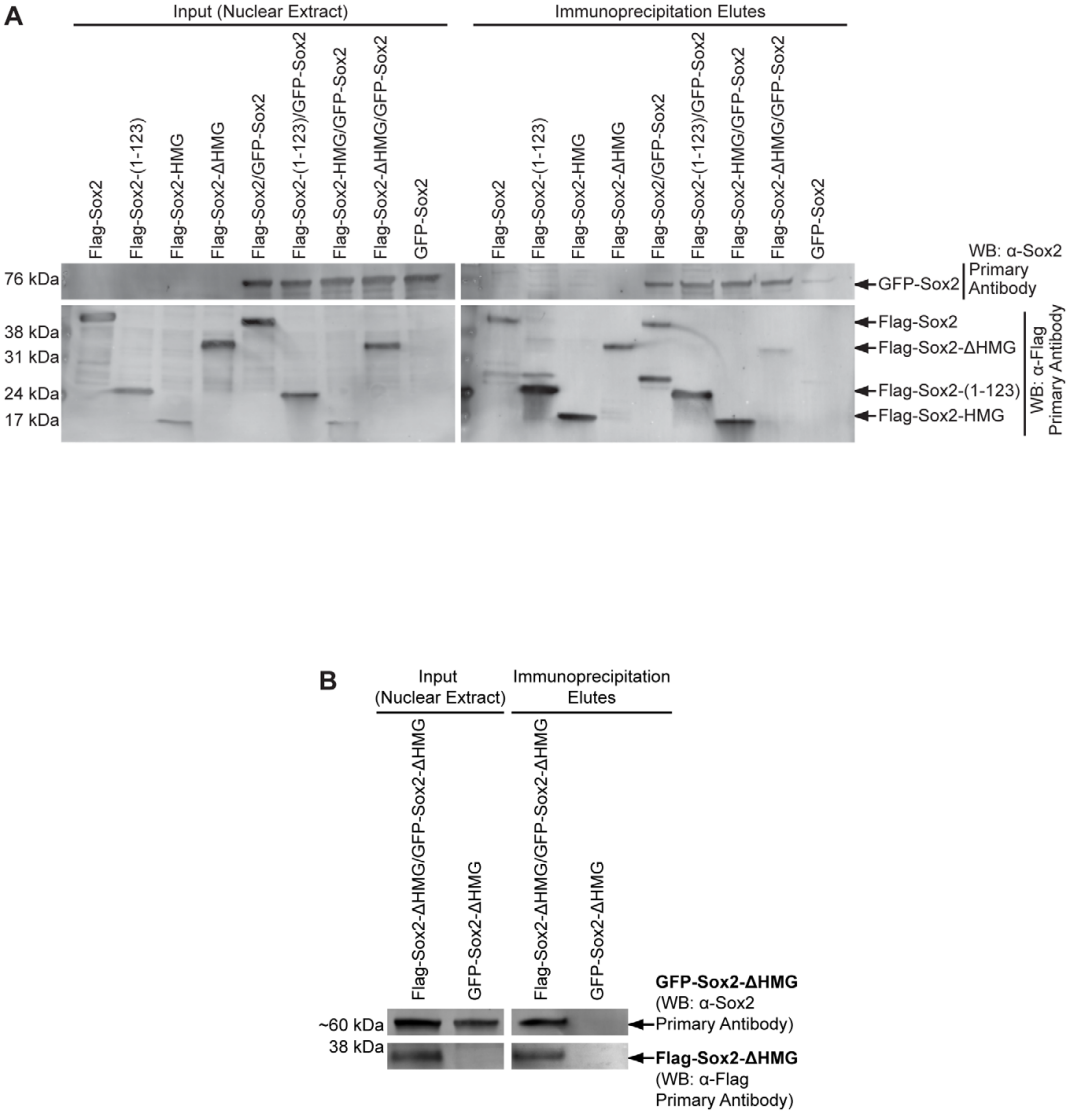
Mapping domains necessary for Sox2 to mediate its self-association. (A) 293T cells were transiently transfected with expression constructs for GFP-Sox2 and the Flag-Sox2 constructs indicated. Nuclear extracts (input lanes) used for co-immunoprecipitation of Flag-Sox2 constructs and associated proteins are presented as western blot analyses (left panels). α-Sox2 was used to visualize GFP-Sox2 (top-left), and α-Flag was used to visualize the Flag-Sox2 constructs indicated. M2-beads were used to co-immunoprecipitate Flag-Sox2 constructs and their associated proteins. Immunoprecipitation eluates were used in western blot analyses (right panels), and probed for GFP-Sox2 (α-Sox2, top-right) or the Flag-Sox2 proteins (α-Flag, bottom-right). (B) Co-immunoprecipitation of GFP-Sox2-ΔHMG by Flag-Sox2-ΔHMG. Experimental design was the same as described for Figure 5A. These experiments were repeated multiple times, and similar results were observed.

Several studies have shown that Sox proteins can form homo- and heterodimers [19]-[21]. In the case of Sox2, we have recently shown that Sox2 can associate with Sox21 [15] and with Sox11 (Cox and Rizzino, unpublished results). Other studies have demonstrated that Sox2 and GFP-tagged Sox2 can associate with one another in nuclear extracts [22]. However, the domains used by Sox2 to self-associate had not been examined. Therefore, we examined the domains used by Sox2 for self-association. We initiated these studies by using two different, N-terminal epitope-tagged versions of Sox2: Flag-Sox2 and GFP-Sox2, which we had previously shown do not adversely affect the function of Sox2 [9], [23]. As in the earlier study [9], use of these constructs enabled us to distinguish Flag-Sox2 from GFP-Sox2 on the basis of size. Using Flag-Sox2 and GFP-Sox2 that were ectopically expressed in 293T cells, we confirmed that Sox2 is able to associate with itself. More specifically, we determined that Flag-Sox2 and GFP-Sox2 are co-immunoprecipitated with M2-beads (Figure 5A). Importantly, Flag-Sox2 did not pulldown GFP (data not shown). The Flag-Sox2 deletion constructs described above were also used to co-immunoprecipitate GFP-Sox2. Remarkably, all mutants tested were able to pulldown GFP-Sox2 (Figure 5A). Furthermore, the Flag-Sox2-HMG construct is sufficient to pulldown GFP-Sox2. This suggested that the HMG domain alone is capable of mediating self-association, though the HMG domain may not be absolutely required for this association.

To determine whether domains of Sox2 other than the HMG are capable of promoting Sox2 self-association, we co-expressed two additional Sox2 proteins, Flag-Sox2-ΔHMG and a GFP-Sox2-ΔHMG, in 293T cells. Flag-Sox2-ΔHMG was able to pulldown GFP-Sox2-ΔHMG, arguing that the HMG domain is not necessary for Sox2 self-association (Figure 5B). As a control, GFP-Sox2-ΔHMG was not immunoprecipitated by M2 Flag affinity beads alone. Thus the HMG domain of Sox2 can promote, but is not required for, Sox2 self-association. Moreover, these data argue that Sox2 is capable of self-association through multiple domains.

## Discussion

Master regulators, such as Sox2, associate with co-activators and co-repressors to precisely bind and regulate their *bona fide* target genes [1], [15]. Moreover, differences in the composition of master regulator-protein complexes undoubtedly influence the transcriptional activity of their target genes. Size fractionation of Sox2 strongly suggests that Sox2 is present in multiple protein complexes, specifically in complexes ranging in size from ∼40 kDa (free Flag-Sox2) to >800 kDa (Flag-Sox2-associated with many proteins simultaneously). In contrast, the size distribution of Sall4 and Lin28 is distinct from that of Sox2, as well as from one another. Thus, it is likely that Sox2-Lin28 protein complexes are disparate from Sox2-Sall4 protein complexes, though complexes containing Sox2, Lin28 and Sall4 may exist in low abundance. This segregation of Sox2 associated proteins into complexes of different molecular weight suggests that different protein complexes are involved in regulating distinct biological functions. For example, high-molecular weight Sox2-protein complexes involved in transcription regulation likely contain Sall4, HDAC1 and other transcription machinery components. Conversely, low-molecular weight Sox2-protein complexes, such as those containing Sox2 and Lin28, may not be involved in transcription, and thus, do not contain additional molecular machinery.

The heterogeneity of Sox2-protein complexes, as well as other master regulator-protein complexes, likely reflects the diverse physiological roles of these complexes. More specifically, ChIP-Chip and ChIP-Seq studies have identified Sox2, and its partner proteins such as, Sall4, Oct4 and Nanog, bound to both active and inactive promoter/enhancer gene regulatory regions [6], [24]–[26]. Recently, identification of Sall4 target genes in ESC have demonstrated that Sall4 co-occupies many Sox2 target genes [24], [25], and our observation that high molecular weight Sox2-protein complexes contain Sall4 reinforces the presence of target genes co-occupied by Sall4 and Sox2. Additionally, the presence of HDAC1, a component of repressor complexes such as NuRD [27], in Sox2-protein complexes may contribute to transcriptional repression of a subset of Sox2 target genes in ESC. In this regard, we have shown that Sox2 and HDAC1 both associate with a putative enhancer of the Sox21 gene in ESC [28]. Thus, it is likely that different Sox2-protein complexes participate in a diverse range of cellular activities.

Interestingly, many Sox2 partner proteins have important biological roles outside of transcription. Our laboratory previously investigated the known ontological functions of Sox2 partner proteins, and found that these proteins participate in biological processes including DNA processing, chromatin organization and assembly, and interestingly, RNA processing [15]. Sox2 associates with a number of RNA processing proteins, including Lin28, Msi2, and Rbm9. However, it is unclear what physiological role Sox2 could play in RNA processing. Lin28 disrupts the processing of let-7 miRNA in both the cytoplasm and nucleus [29], [30]. It is possible that Sox2-Lin28 protein complexes may mediate a variety of biological process in the nucleus, potentially including the processing of let-7 miRNA. Further studies are needed to elucidate the role of Sox2, and its associated protein complexes, in these diverse biological functions.

**Figure 6:**
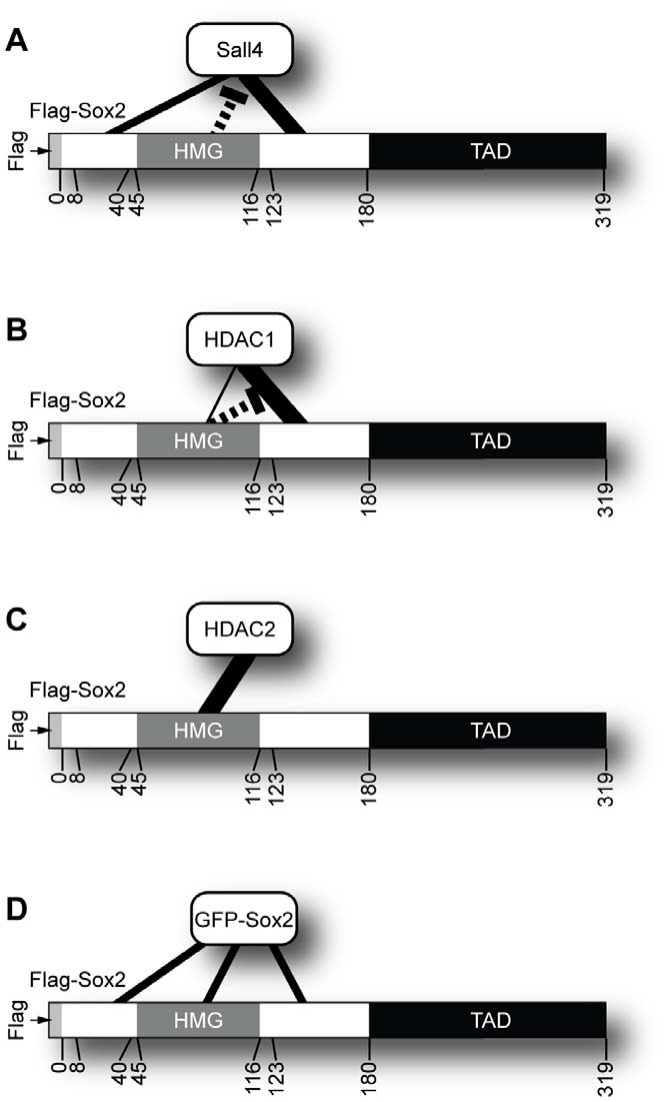
Model of Sox2-protein associations. (A) Sall4 associates with Sox2 primarily through a region C-terminal to the HMG domain of Sox2. The region N-terminal of the HMG domain of Sox2 also mediates the association between Sox2 and Sall4; whereas, the HMG domain of Sox2 may weakly interfere with the association between Sox2 and Sall4, as indicated by the dashed line. (B) HDAC1 primarily associates with Sox2 through Sox2 amino acids 124 to 180. The dashed line indicates interference of association observed between Flag-Sox2 and HDAC1 by the Sox2-HMG domain. (C) Endogenous HDAC2 (from 293T cells) primarily associates with Flag-Sox2 through the HMG domain of Sox2. (D) Flag-Sox2 associates with GFP-Sox2 through multiple domains.

Our efforts to map the domains used by Sox2 to associate with other proteins argue that specific, and sometimes, multiple domains of Sox2 are used to associate with its partner proteins (Figure 6). More specifically, a domain C-terminal to the HMG domain, and to a lesser extent, a domain N-terminal to the HMG domain of Sox2, appear to promote its association with Sall4 (Figure 2B, 6A); whereas, Sox2 appears to associate with HDAC1 primarily through a domain located within the amino acid region 124–180 (Figure 3A, 6B). Interestingly, the HMG domain, in the context of full-length Sox2, appears to interfere with its association with HDAC1, though the HMG domain alone is capable of co-immunoprecipitating HDAC1 (Figure 3B). Currently, it is unclear how the HMG domain of Sox2 modulates its association with HDAC1. Surprisingly, the HMG domain of Sox2 is principally responsible for mediating the association between Sox2 and HDAC2 (Figure 4, 6C).

The work described in this study did not address whether the association of Sox2 with Sall4, HDAC1 or HDAC2 is direct or indirect. We attempted to address this question for the self-association of Sox2 using Sox2 proteins produced by *in vitro* transcription/translation. Specifically, Flag-Sox2-(1-180) and Sox2 were produced *in vitro*, and tested for their ability to be co-immunoprecipitated using M2-beads. Although the proteins were produced successfully, as determined by western blot analysis, co-immunoprecipitation of Sox2 by Flag-Sox2-(1-180) was not detected (data not shown). It is possible that *in vitro* transcribed/translated Sox2 does not self-associate for a number of reasons, including: the protein may not fold properly when produced by *in vitro* transcription/translation. We suspect that this is unlikely for two reasons. First, the crystal structure of the N-terminal domain of Sox2 was determined using recombinant Sox2 (amino acids 1-123) [17]. Second, full-length Sox2 protein is able to refold and bind to DNA after being heated to 90°C [31]. Alternatively, Sox2 self-association may require proper post-translational modification. In this regard, Sox2 has been shown in different studies to be acetylated, phosphorylated, sumoylated and/or poly-(ADP)-ribosyoated *in vivo* [18], [32]–[35]. Lastly, our experimental approach may not have had the necessary sensitivity to detect the low abundance of co-immunoprecipitated Sox2.

Although we did not determine whether Sox2 is able to self-associate by direct interaction, our studies do indicate that Sox2 uses several domains to promote its self-association (Figure 6D). The HMG domain of Sox2 is capable of mediating Sox2 self-association, though the HMG domain is not absolutely required for self-association (Figure 5B). Moreover, the observation that the HMG domain is sufficient for Sox2 self-association suggests that HMG domains of distinct Sox proteins, which are highly conserved, may be able to promote Sox protein association. In this regard, our laboratory recently identified Sox21 as a Sox2 partner protein in our unbiased proteomic screen [15]. Additionally, we have determined that Sox2 can associate with Sox11 (Cox and Rizzino, unpublished results). Furthermore, other studies suggest that the association between Sox proteins and their self-association is critical for proper development. Previous reports have demonstrated that several Sox proteins, including Sox8, Sox9 and Sox10, are capable of forming homo- and heterodimers [19], [20]. During chondrogenesis, expression of the *col2A1* locus relies heavily upon Sox protein homo- and heterodimers for proper expression. Sox9, which uses a DNA-dependent dimerization domain common to Group E Sox proteins, binds to the *col2A1* locus as a dimer [20]. Additionally, Sox5 and Sox6 use coiled-coil DNA-independent dimerization domains to associate together with the *col2A1* locus as well [21].

In conclusion, we demonstrate that Sox2 uses several different domains to associate with several other ESC proteins. Additionally, we demonstrate that Sox2 is present in protein complexes that vary widely in size. The observation that Sox2 and other master regulators interact with a diverse library of co-activators and co-repressors adds another level of complexity to our efforts to understand transcriptional regulation in ESC. Through this diversity, Sox2 and its partner proteins are able to precisely control both the expression of the gene networks necessary for the proper maintenance of ESC, as well as the programs necessary to direct differentiation and development.

## Materials and Methods

### Cell culture and transient transfection

Cultivation of i-Sox2-ESC and 293T cells, and transient transfection of 293T cells have been described previously [16], [36].

### Nuclear extract preparation from i-Sox2-ESC and size fractionation of nuclear proteins

The preparation of nuclear extracts from doxycycline (dox)-induced and uninduced i-Sox2-ESC by Dounce homogenization has been described previously [15], [37]. Nuclear extracts prepared by Dounce homogenization were used for size fractionation of Flag-Sox2-, Sall4-, and Lin28-protein complexes using SuperdexTM-200 (GE Healthcare, Piscataway, NJ) chromatography. Chromatographic fractions were concentrated using TCA precipitation as described previously [15].

### Plasmid constructs

The expression vectors for Flag-Sox2, Flag-Sox2-ΔHMG, Flag-Sox2-(1-180), GFP-Sox2, GFP-Sox2-ΔHMG and Sall4 have been described previously [15], [23]. The mouse HDAC1 expression plasmid was obtained from Open Biosystems (Clone ID: MMM1013-98478099) (Open Biosystems, Huntsville, AL). The expression vector for Flag-Sox2-(1-123) was generated by insertion of the coding sequence for amino acids 1 to 123 of Sox2 with an N-terminal Flag epitope into the mammalian expression vector pCMV5. This was accomplished by PCR amplification of the required sequence from the Flag-Sox2 expression plasmid. The sequences of primers used for generating Flag-Sox2-(1-123) were:

Flag-Sox2-(1-123)-F: ggcgaattg**GGTACC** ***GCCACCATGGACTAC***

Flag-Sox2-(1-123)-R: tcctttt**TCTAGA**TTATTATTACTTCATGAGCGTCTTGG

Uppercase, bold, underlined sequence in Flag-Sox2-(1-123)-F and Flag-Sox2-(1-123)-R primers refer to KpnI and XbaI restriction sites, respectively. Lower case sequences at the 5′ end of Flag-Sox2-(1-123)-F and Flag-Sox2-(1-123)-R primers refer to overhangs that facilitate cleavage of free DNA ends by KpnI and XbaI restriction enzymes, respectively. Uppercase, bold, italicized, and underlined sequence in Flag-Sox2-(1-123)-F primer refers to partial Flag epitope tag sequences. Uppercase, bold, italicized sequence in Flag-Sox2-(1-123)-F primer refers to Kozak sequence. Unmodified sequence in Flag-Sox2-(1-123)-R primer refers to Sox2 sequence ending with three stop codons. The PCR product was digested with KpnI and XbaI restriction enzymes, and ligated into pCMV5 vector that had been digested with the same enzymes.

To generate Flag-Sox2-HMG expression plasmid, site-directed mutagenesis was performed using Flag-Sox2-(1-123) expression plasmid as template to introduce an RsrII restriction site at the end of the nucleotide sequence coding for the amino acid residue 7 of the Sox2 protein. The PCR product was digested with DpnI to remove the template plasmid, digested with RsrII restriction enzyme to remove the sequence coding for amino acids 8-40, and ligated to generate Flag-Sox2-HMG expression plasmid. The sequences of primers used for generating Flag-Sox2-HMG expression plasmid are listed below:

Flag-Sox2-HMG-U: GAACAGCCCGGACCGCGTCAAG

Flag-Sox2-HMG-L: GCGGCTTC***CGGTCCG***TCTCCATC

Bold, italicized, and underlined sequences in Flag-Sox2-HMG-L primer refers to bases changed to introduce an RsrII restriction site. Bold, italicized sequence in Flag-Sox2-HMG-L primer refers to the RsrII restriction site.

### Nuclear extract preparation from 293T cells, immunoprecipitation and western blot analysis

One day after transfection, cells were harvested, nuclear extracts were prepared using a NE-PER™ kit (Pierce, Rockford, IL), and protein concentration of the nuclear extracts were determined using Micro BCA™ Protein Assay Kit (Pierce). For immunoprecipitation, equal amounts of nuclear protein for various conditions examined were added to M2-beads (Sigma-Aldrich, St. Louis, MO) to isolate Flag-tagged protein complexes, as described previously [36]. Equal amounts of nuclear protein (input) and equal volumes of immunoprecipitate eluates, were separated by SDS-PAGE, and transferred to PVDF membrane (Millipore, Billerica, MA). Blots were blocked in 5% (w/v) non-fat milk in TBST (0.1% (v/v) Tween), and incubated in primary antibody followed by secondary antibody. The western blots were developed using ECF substrate (GE Healthcare, Piscataway, NJ), and the fluorescent signal was scanned using a Typhoon™ 9410 Variable Mode Imager (GE Healthcare, Piscataway, NJ). For immunoblotting of Sox2, we used a Sox2 antibody (ab15830, Abcam, Cambridge, MA) at a dilution of 1∶1,000 and a secondary α-rabbit AP conjugate (A3687, Sigma-Aldrich) at a dilution of 1∶10,000. For immunoblotting of Flag-tagged Sox2 proteins, we used an α-Flag antibody (F1804, Sigma-Aldrich) at a dilution of 1∶1,000 and a secondary α-mouse AP conjugate (A4312, Sigma-Aldrich) at a dilution of 1∶10,000. For immunoblotting of Sall4, we used a Sall4 antibody (sc-46045X, Santa Cruz Biotechnology, Santa Cruz, CA) at a dilution of 1∶5,000 and a secondary α-goat AP conjugate (A4187, Sigma-Aldrich) at a dilution of 1∶15,000. For immunoblotting of HDAC1, we used an HDAC1 antibody (ab7028, Abcam) at a dilution of 1∶5,000 and a secondary α-rabbit AP at a dilution of 1∶10,000. For immunoblotting of HDAC2, we used a HDAC2 antibody (ab7029, Abcam) at a dilution of 1∶5,000, and a secondary α-rabbit AP conjugate at a dilution of 1∶10,000. For immunoblotting of Lin28, we used a Lin28 antibody (ab46020, Abcam) at a dilution of 1∶5,000, a secondary α-rabbit AP conjugate at a dilution of 1∶10,000.
